# Diflunisal compassive use in transthyretin familial amyloidotic polyneuropathy (TTR-FAP): report of the first Spanish experience

**DOI:** 10.1186/1750-1172-10-S1-P2

**Published:** 2015-11-02

**Authors:** Sebastián E Azorín Contesse, Christopher Cabib, Josep M Campistol

**Affiliations:** 1 Hospital Clínic de Barcelona, Amyloidosis and Monoclonal Gammopathies’ Unit (UDAM); Nephrology and Transplant Unit (SNiTR), 08036, Barcelona, Spain; 2 Hospital Clínic de Barcelona, Electromyography, Motor Control and Neuropathic Pain Unit, Neurology Service, 08036, Barcelona, Spain

## Background

Diflunisal is a well known FDA-registered commonly used NSAID therapy in the USA since the 1970's. In Europe, the drug has been seldom authorised on a national basis with the next safety update report scheduled for 2025 (http://www.ema.europa.eu/ema/). Spain is one such country where commercial use has not been authorised, likely because of concerns on liver hypersensitivity and availability of other NSAIDs. Interestingly, recent advances have shown a potential beneficial effect in transthyretin (TTR) hereditary amyloidosis, as evidenced by encouraging data from the diflunisal trial consortium (Berk et al. JAMA. 2013;310(24):2658-2667) where quality of life, neuropathy impairment scores and nutritional status showed significant, though modest, better results in patients randomised to receive diflunisal instead of placebo.

## Methods

We aimed at describing the first off-label (compassive) use of diflunisal in a small cohort of 10 patients affected by variable degrees of TTR-FAP in our centre. A protocol for off-label use Diflunisal was introduced and accepted early in 2014 by the amyloidosis and monoclonal gammopathies’ unit (UDAM) at Hospital Clínic de Barcelona. Inclusion criteriae consisted of any symptomatic hereditary TTR-amyloidosis patient with progression of FAP either (i) unfit or unwilling to receive either liver transplantation (LT)/Vyndaqel® as per on-label indication or to enter an ongoing clinical trial (e.g. RNA silencing), (ii) already under on-label treatments (Vyndaqel®, liver transplantation (LT)) or (iii) any Domino-LT (DLT) recipient with de novo signs/symptoms of polyneuropathy with biopsy proven culprit ATTR deposits. Exclusion criteriae consisted of a known previous adverse reaction to Diflunisal, estimated GFR <60mL/min/1.73m2, concommittant lithium therapy, renin-angiotensin-aldosterone system antagonists, being already under investigational drug or tafamidis, Karnofsky score <40 and unwillingness to comply to follow-up. Basal demographic and clinical characteristics were collected. Follow-up was performed every 2 months with particular attention to disease progression “red flag” signs (polyneuropathy, dysautonomy, ECG) and incidence of adverse events.

## Results

A total of 10 patients were included. After a median follow-up of 8 months, diflunisal showed overall improvement of neuropathic pain and quality of life as well as stabilisation of disease stage. Mean initial eGFR was 83 and did not change significantly. Three cases presented transient acute renal failure that recovered once diflunisal dose was lowered, without a detrimental effect on TTR-FAP.

## Conclusion

This is the first Spanish report of diflunisal off-label use in the setting of hereditary TTR-FAP. Diflunisal seems a relatively safe and effective option for patients with TTR-FAP in progression and who are not candidates for other therapies. Particular attention must be paid to renal function as dose adaptation may be warranted.

